# Influence of Autologous *In Vitro* Activation of Ovaries by Stem Cells and Growth Factors on Endocrine and Reproductive Function of Patients with Ovarian Insufficiency-A Clinical Trial Study 

**DOI:** 10.22074/IJFS.2020.134678

**Published:** 2021-06-22

**Authors:** Suada Tinjić, Džihan Abazović, Dušica Ljubić, Danilo Vojvodić, Tatjana Božanović, Mirza Ibrišimović, Sergije Marković, Aleksandar Ljubić

**Affiliations:** 1 Department of Gynecology, Polyclinic, Korak do Života, Tuzla, Bosnia and Herzegovina; 2 Department of Regenerative Medicine, Remedika Hospital, Belgrade, Serbia; 3 Department of Gynecology, MediGroup Hospital, Belgrade, Serbia; 4 Faculty of Medicine, Military Medical School, Belgrade, Serbia; 5 Department of Gynecology and Obstetrics, Medical School, University of Belgrade, Belgrade, Serbia; 6 Department of Medical Biology and Human Physiology, Sarajevo Medical School, University Sarajevo School of Science and Technology, Sarajevo, Bosnia and Herzegovina; 7 Department of Histology, Medical School, University of Tuzla, Tuzla, Bosnia and Herzegovina; 8 Department of Gynecology, Dubrovnik International University, Dubrovnik, Croatia

**Keywords:** Growth Factors, Ovarian, Premature Ovarian Failure, Stem Cells

## Abstract

**Background:**

Premature ovarian failure (POF) can be found in 1% of women at the age of 35-40, mostly due to unknown causes. PI3K-Akt signaling is associated with both ovarian function and growth of primordial follicles. In this study, we examined the effects of autologous *in vitro* ovarian activation with stem cells and autologous growth factors on reproductive and endocrine function in patients with ovarian impairment.

**Materials and Methods:**

The longitudinal prospective observational study included 50 patients (between 30 and 50 years) with a diagnosis of POF and infertility. This multicenter study was performed at Jevremova Special Hospital in Belgrade, Saint James Hospital (Malta), and Remedica Skoplje Hospital, between 2015 and 2018. All patients went through numerous laboratory testings, including hormonal status. The autologous bone marrow mesenchymal stem cells (BMSCs) and growth factors were used in combination for activation of ovarian tissue before its re-transplantation. The software package SPSS 20.0 was used for statistical analysis of the results.

**Results:**

Differences in follicle stimulating hormone (FSH), luteinizing hormone (LH), estradiol (E2), and progesterone (PG) hormone concentrations before and after 3, 6, and 12 months post-transplantation were tested in correlation with the volume of transplanted ovarian tissue. A significant correlation (P=0.029) was found between the change in E2 level after 3 months and the volume of re-transplanted tissues. Also after re-transplantation, 64% of the patients had follicles resulting in aspiration of oocytes in 25% of positive women with follicles.

**Conclusion:**

The SEGOVA method could potentially solve many human reproductive problems in the future due to the large number of patients diagnosed with POF, as well as the possibility of delaying menopause, thus improving the quality of life and general health (Registration number: NCT04009473).

## Introduction

Female gametes are extremely sensitive cells in the human body, which decrease in number drastically from fetal period to the prepuberty when they count about 300 000. Their number continues to decrease in life, so that the residual pool in women over 35 years of age is about 25 000 oocytes (1). Early folliculogenesis begins autonomously and independently from follicle stimulating hormone (FSH). Inactive primordial follicles form an ovum reserve that is continually reduced by aging, genetic factors, drugs and surgery. 

Follicles show initial growth but end with atresia. Those which continue to grow and develop further become preantral, and when they achieve 3-6 layers of granulosa cells, cell mobilization from the ovary stroma begins, and the cells gradually group in the outer sides of the basal membrane. This event simply does not occur if the oocyte does not secrete biosignals, mainly the differentiating growth factor 9 (GDF-9) (2). 

Peptide hormones of granulosa cells are important paracrine modulators of theca cells, and also affect the functions of gonadotropic pituitary cells. These effects in the middle of the follicular phase lead to the selection of a dominant follicle and formation of a preovulation follicle. Antimüllerian hormone (AMH), with its paracrine action, inhibits the FSH effect, and together with the pituitary gland, reaches its maximum production on the seventh day of the follicular phase, selectively inhibits the release of FSH and leads to atresia of the competitive follicle (3). 

From 300,000 follicles at the beginning of puberty, 450 of them achieve the ovulation phase, while about 660 follicles are consumed for one ovulation. In each ovary we normally find 5-15 antral follicles called antral follicular count (AFC), which is correlated with both the level of AMH and the concentration of inhibitors B, in particular (4). In 1967, De Moraes Rueshen and Jones were the first to set the definition of primary premature insufficiency of the ovary (POF) as a non-physiological interruption of menstruation after puberty (1). POF is a syndrome recognized by hypergonadotropic amenorrhoea and hypoestrogeny. There are questions arising about possible existence of a common pathway that controls egg cell atresia or the possibility of its activation by different gene mutations (5, 6). The medical treatment of POF patients should include the following aspects: ovarian hormone replacement, fertility restoration, and improvement of the patient's psychological aspect as a prevention of complications arising from loss of ovarian function that affect the quality of life.

The SEGOVA is a group of procedures and methods designed by Ljubić et al. (7) to improve women’s general and reproductive health by restoring ovarian function and improving the quality of life. This method offers minimally invasive approaches including ovarian cortex laparoscopic biopsia and retransplantation to the same site from which the tissue was taken for *in vitro* activation. The second laparascopic surgery is avoided together with chemical stimulation of genetic pathways due to application of autologous platelet rich plasma (PRP) growth factors. This novel approach involves application of bone marrow stem cells (BMSC) that can be readily obtained and amplified in large quantities *in vitro*. BMSCs are a good choice for transplantation in patients with POF. BMSCs migrate to the impaired ovary and together with cytokines that have antiapoptotic, antifibrotic, anti-inflammatory and immunoregulatory effects improve ovarian function (8, 9). 

The results of this novel study indicate that the SEGOVA procedure can play a potentially important role in improving endocrine ovarian function in patients with impaired or lost ovarian function.

## Materials and Methods

### Study design and selection of respondents

In this longitudinal prospective observational study, we included 50 patients in the age of 30 to 50 years with a diagnosis of POF and infertility (Clinical Trials.gov Identifier: NCT04009473). The study was performed at Jevremova Special Hospital in Belgrade, Sant James Hospital in Malta, and Remedica Skoplje Hospital, between 2015 and 2018. The criteria for patient involvement included the following: signed consent form, women over 18 years of age, primary or secondary amenorrhea for at least 3 months, AMH hormone values <0.42 ng/ml and FSH >20 IU/L, and/or failure of previous attempts at assisted reproductive techniques due to limited ovarian response (less than 4 oocytes obtained), normal karyotype 46, XX, presence of at least one ovary, normal anti-microsomal antibodies, and proper thyroid function as evidenced by normal serum levels of thyroid stimulating hormone (TSH). The exclusion criteria included: currently pregnant or breastfeeding, history or evidence of existing gynecological malignancy, the presence of adnexal masses indicating the need for further evaluation, mental health disorder, active substance abuse or addiction, existence of contraindication to laparoscopic surgery and/or general anesthesia in the past two weeks, use of the following medicines: oral or systemic corticosteroids, hormones (estrogen, progestin, oral contraceptives), Danazol, anticoagulants, herbal or botanical supplements with possible hormonal effects, type I or type II diabetes mellitus or if they are receiving antidiabetic medicines, known significant anemia (hemoglobin <8 g/dL), deep vein thrombosis and/or pulmonary embolus, cerebrovascular disease, presence of heart and liver disease [defined as aspartate aminotransferase (ASt) or alanine aminotransferase (ALT)> 2 times normal, or total bilirubin> 2.5 mg/dL], and presence of kidney disease (defined as blood urea> 30 mg/ dL or serum creatinine> 1.6 mg/dL). In addition, patients with positive history of cancer, in case of any positive tumor markers, or with structural changes on the ovaries, would be immediately excluded from the procedure. However, no such patients applied for this study.

Before the procedure, patients had to have balanced nutritional status, optimized trace elements and antioxidants through proper nutrition and supplements, as well as optimized physical activity (exercise plan).

### Preparation for the SEGOVA procedure

All patients went through the following laboratory testing:

1. (Hormonal status, Ca-125, Ca-19, Ca-19,9:HE-4, ROMA index, Beta HCG, AFP, SE, CRP);
2. Karyotype;
3. Microbiological analysis of vaginal and cervical swabs with antibiogram, cervical swabs on Ureoplasm, Mycoplasma and Chlamydia) - not older than 3 months;
4. Kg Rh D factor, total blood cell count, blood glucose, urea, creatinine, K, Na, NaHCO, proteinogram, coagulogram;
5. Serological analysis: HIV, HBSAg, HCV;
6. Lung RTG, findings and opinion of internal medicine doctors for general anesthesia;
7. 3D ultrasound (presence of antral follicles and their number, ovarian volume), personal and family history, length of infertility, number of *in vitro* fertilization (IVF) cycles.

All patients answered questions about menstruation, parity, the prevalence of POF in the family, education, and possible pre-amenorrhea stress, and a quality of life questionnaire. In addition, all women diagnosed with POF included in this study had normal results for the following: blood count, glucose, and lipoprotein profile, prolactin, testosterone, androstenedione, DHEA sulfate, 17-OH progesterone, thyroxine, TSH, parathyroid hormone, adrenocorticotropic hormone and cortisol. All tested patients had negative test results for anti-ovarian antibodies, anti-cardiolipin antibodies, anti-thyroglobulin antibodies, anti-microsomal antibodies, and had a normal karyotype. Sonographic examination of the pelvis in all patients showed that there were no pathological changes in the ovarian or follicular activities. All women included in this study signed a written consent approved by the Ethic Committee of Jevremova Special Hospital for Gyneclogy in Belgrade, Serbia (number: 63/2015).

### The SEGOVA method

The SEGOVA is a group of procedures and methods designed by Professor Aleksandar Ljubić to improve women’s general and reproductive health by restoring ovarian function and improving the quality of life. The SEGOVA approach has several important advantages over other in vitro ovarian activation approaches

1. First, conservative minimally invasive surgery (ovarian cortex laparoscopic biopsy). This approach permits re-transplantation to the same site, from which the tissue was taken for *in vitro* activation.
2. Autologous platelet-rich plasma (PLRP) growth factors are used instead of chemical stimulation of genetic pathways.
3. Second laparoscopic surgery is avoided, *in vitro* activated ovarian tissue is re-transplanted through ultrasound control.

### The procedure consists of several stages:

1. Laparoscopic ovary tissue biopsy (the isolated ovarian tissue was sent for pathohistological analysis and none of the samples were positive for malignancy);
2. Under laboratory conditions, micro-fragmentation inhibits a group of HIPPO genes that adversely affect follicular growth.
3. Autologous activation is then performed by the growth factors of the AKT group of genes that positively influence the growth and development of the follicles;
4. Bone marrow biopsy and MSC processing;
5. Transplantation of activated ovarian tissue under the control of 3D doppler ultrasound into the ovary;
6. Bone marrow stem cell implantation into ultrasound-controlled ovarian tissue;
7. Activated tissue has an effect on the cells, which triggers the function of hormone production, follicular growth, and the activation and differentiation of oocytes. The hormonal status of the patients were always assessed on days 2-5 of menstrual cycle.

### Description of SEGOVA procedure and isolation of stem cells

The procedure of surgical resection of the ovarian cortex is performed using a modified "single port" laparoscopic technique with an entrance through the umbilicus. After an incision of about 2 cm in the umbilical zone, three 5 mm portals will be placed, establishing an intra-abdominal pressure between 10 and 12 mm Hg. A 5 mm diameter laparoscope is then introduced, as well as auxiliary trocars. In some cases, and as an aid to the technique, a uterine manipulator is introduced transvaginally. After visualization of the ovaries, the cortex is fixed with the help of adequate instruments and cut with another instrument (scissors). Hemostasis control, port removal and wound closure are performed subsequently.

Further, the obtained ovarian cortices are chopped by multiple cuttings with a scalpel no. 25 to fragments that are smaller than 1×1 mm. The tissue prepared in this way is weighed using an analytical balance and placed in a petri dish, where it is washed with gamete buffer (Cook). After washing, tissue samples are transferred to PRP medium and further activated by autologous thrombin medium (Sigma-Aldrich). The sample prepared in this way is incubated for 48 hours at a temperature of 37°C and in 5% CO_2_ . In addition, under ultrasound monitoring with a 3D color ultrasound (GE Voluson 730 Pro), a transvaginal puncture of both ovaries is performed under general anesthesia, with a 30 cm long 16 gauge needle. After incubation, fragmented ovarian tissue with PLRP is injected into the subcortical region of the right and left ovaries (2.3 ml and 2.1 ml, respectively).

Bone marrow cells are taken from the tibia or iliac bone. The biopsy is performed under general or local anesthesia. A small incision (7 mm) is made with a special needle to penetrate the periosteum. A volume of 100 to 150 ml of bone marrow is aspirated and centrifuged in a particularly automated Angel system-Arthrex under sterile conditions. After extraction, the samples were centrifuged to separate the acellular part and the erythrocytes which were discarded from the nucleated cells. Nucleated cells were further used for treatment. After treatment, 3-5 mL of bone marrow aspirate concentrate (BMAC) is obtained. Bone marrow concentrate contains a significant number of stem cells, leukocytes, platelets, erythrocytes, hematopoietic stem cells (HSCs), and MSCs. Each sample was analyzed by flow cytometry using specific markers: CD34, CD146, CD90, CD45, CD105, CD73, CD133, Stro-1. Cell viability and the total nucleated cell count (TNCs), were assessed using 7AAD. The ovarian tissue is microfragmented, incubated and activated by autologous growth factors. After incubation, the prepared tissue is mixed with BMAC and re- transplanted via intraovarian injection.

### Platelet rich plasma preparation

The platelet-rich plasma (PRP) procedure involves drawing blood from patients (104 ml) with a special double syringe, which is then left to be centrifuged at the appropriate number of revolutions over a period of time to separate plasma-rich growth factors. A separate syringe enriched with platelets is then drawn with another syringe and used for further treatment. The whole blood will be processed using the Angel separation system (Arthrek, USA). Separation of PRP from lowplatelet plasma is performed in a closed system - in a fully automated machine designed for this purpose. This machine can process from 40 ml to 180 ml of blood taken from the patient. Under sterile conditions, using the Angel system, 18 times higher the platelet concentration in PRP than the baseline measured value in the patient can be obtained. In our case, the optimal concentration is between 6 and 8 times more concentrated than in patient’s blood, which can be determined and adjusted on the machine. For the PRP procedure and laboratory analysis, a whole blood sample will be taken from the cubital vein and mixed with Anticoagulant Acid Citrate Dextrose (ACD) in a ratio of 7: 1. Two 60 mL syringes will be taken and filled with up to 60 mL blood, making a total volume of 104 mL whole blood and 16 mL ACD. The second phase, or laboratory phase, involves the application of complex technology, which uses special separators and systems, divides and filters certain cells, prepares them and activates the growth factors in them. At the end of this procedure, we get between 5-7 mL of active plasma containing a large amount of growth factors, including PDGF, TGF-β, VEGF, EGF, and many others. In the next step, a volume of PRP product (5 mL) is mixed with the prepared ovarian fragments. Immediately before administration, PRP is activated by autologous thrombin in a 10: 1 ratio. The maximum time interval from activation to the end of instillation in the ovary is 45 seconds. Activation is performed for each ovary separately. 

### Parameter monitoring

The changes in hormone levels in serum of the patients were evaluated 3, 6 and 12 months after the procedure. The effects of hormonal changes on ovarian reproductive function (follicle count, number and quality of aspirated cells, and number of embryos) were monitored using ultrasound and based on the outcome of IVF procedures.

### Statistical analysis

Since the observed sample size of the included women was relatively small, and the data on the level of observed hormones, the volume of ovarian cortex autografted and the volume of the BMAC of the patients were not homogeneous, different methods of nonparametric statistics were used for data analysis, depending on the specific goals of the study. The data was processed in the statistical package SPSS 20.0. To investigate the changes in the levels of the hormones FSH, LH, progesterone (PG) and estradiol (E2), the amounts detected prior to the intervention, as well as 3, 6 and 12 months post-intervention, were compared using Wilcoxon’s rank test. Namely, the following null hypotheses have been tested: median levels of a particular hormone detected before the intervention do not differ from the median hormone levels at 3 months post-intervention, 6 months post-intervention and 12 months post-intervention. The alternative hypothesis is that differences among hormone medians measured at different time points do exist. The hypotheses are being tested using Z statistics, based on the data difference directions, but also the relative strength of these differences. This approach represents one of the most useful non-parametric methods. The test statistic Z is approximately normally distributed with an arithmetic mean equal to zero and a variance equal to one, and has the form of:

$$Z=\frac{W-{µ}_{w}}{\sigma_{w}},$$

where W is a statistic representing a smaller sum of rankings with one character

$$W=\min(W_{-},W+);$$

μ_w_ is the arithmetic mean of the W statistics and is calculated according to the formula:

$${µ}_{w}=\frac{n\left(n+1\right)}{4};$$

a σ_w_ represents the standard deviation of the statistics W and is obtained according to the formula:

$$\sigma_{w}=\sqrt[{}]{\frac{n\left(n+1\right)\left(\mathrm{2n}+1\right)}{24}}$$

In order to investigate the effects of the volume of re-transplanted activated ovarian tissue on changes in hormone values of FSH, LH, PG and E2, Spearman’s coefficient of correlation of the rank was used. The Spearman’s rank correlation coefficient r_s_ measures the dependence between the rankings of two data sets and is obtained according to the formula:

$$r_{s}=1-\frac{6\Sigma_{\mathrm{i=1}}^{n}6d_{i}^{2}}{{n\left(n^{2}-1\right)}^{'}}$$

where d_i_ is the difference between the rankings of two data sets. Certainly, after calculating the value of the Spearman’s rank correlation coefficient, it is necessary to check the statistical significance. Testing is done by checking if the null hypothesis coefficient is not statistically significant (H_0_ :ρ_s_ =0) against the alternative hypothesis that it is statistically significant (H_0_ :ρ_s_ ≠0). The test statistics formula used for this purpose is:

$$t=\frac{r_{s}-{𝜌}_{s}}{\sqrt[{}]{\left(\frac{1-r_{s}^{2}}{n-2}\right)}}$$

### Results

#### Average hormonal levels over twelve months after the intervention

The follow-up of hormones FSH, LH, E2 and PG included evaluation of the changes in values of the observed hormones before the intervention and at three, six, and twelve months post-intervention. 

When observing the pre-intervention period, our results showed that 50% of the women had a FSH hormone value ranging from 28.10 to 86. 50 IU/mL, while the median value of this hormone was 46.50 IU/ mL. These values were obtained on the basis of a sample of 47 women. 

Three months after re-transplantation, the median FSH level was raised (2.68%) compared to the preintervention period and amounted to 47.75 IU/mL, with 50% of the women ranging between 24.28 and 86.50 IU/mL. These values were obtained from 19 women at 3 months following the intervention.

Three months after re-transplantation, the median FSH level was raised (2.68%) compared to the preintervention period and amounted to 47.75 IU/mL, with 50% of the women ranging between 24.28 and 86.50 IU/mL. These values were obtained from 19 women at 3 months following the intervention.

For the period of 6 months after intervention, the median FSH hormone value was 34.49 IU/mL, and 50% of the women had a FSH hormone value between 15.80 and 63.50 IU/mL. These values were obtained from 23 women. At 6 months post-intervention, we noticed that both median values were decreased by 25.82% compared to this value prior to the intervention.

**Fig.1:**
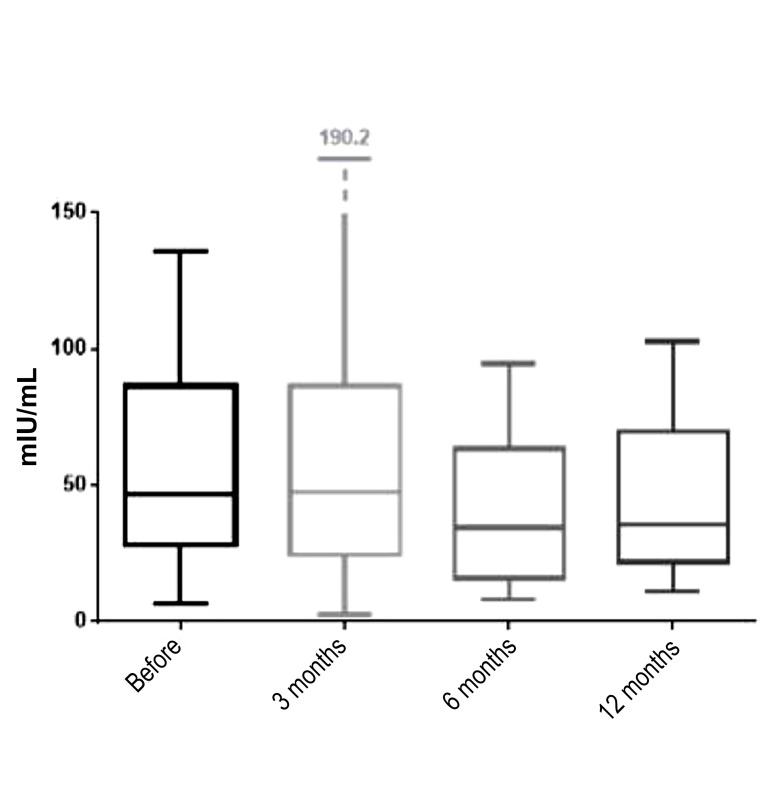
Medians, interquartile range and range of follicle stimulating hormone (FSH) before the procedure and 3, 6, and 12 months after procedure (line inside the box is median; the box is interquartile range; the lines outside the box are minimum and maximum values).

The last follow-up measurement was done at 12 months post-intervention, when the mean FSH hormone value was 35.36 IU/mL (23.95% decrease compared to the preintervention value), while 50% of the women had a FSH level ranging from 21.86 to 70.08 IU/mL. Due to the loss of the follow-up data in some of the patients at 12 months after the intervention, the results were obtained on a sample size of 14 women. The median values of the FSH hormone before intervention and at 3, 6 and 12 months after the procedure, are shown in Figure 1. 

**Fig.2:**
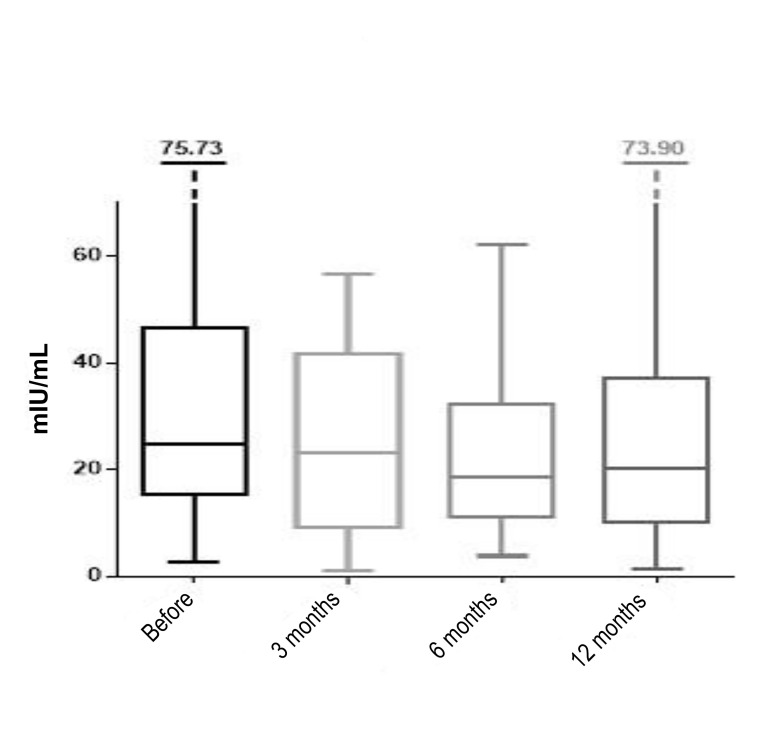
Medians, interquartile range and range of luteinizing hormone (LH) before the procedure and after 3, 6, and 12 months (line inside the box is median; the box is interquartile range; the lines outside the box are minimum and maximum values).

Before the intervention the median value of the hormone LH was 24.80 mIU/mL, based on the measurement of the hormone value in 45 women. The median LH value was slightly decreased (7.29%) after 3 months and was 22.99 mIU/mL. At 6 months post-intervention it further decreased to 18.60 mIU/mL median value (25% decrease compared to before the intervention). After twelve months the median value was 20.19 mIU/mL, which is 18.58% reduction compared to the value prior to the intervention. These values were obtained from sample sizes of 20, 21, and 16 at 3, 6 and 12 months, respectively. We can also observe the change in the borderline interval values of hormone LH, resulting from 50% of the women. Before the interventions the boundaries of this interval ranged from 15.22 to 46.56 mIU/mL, while 3 months after the intervention the boundaries were 9.23 to 41.73 mIU/mL, from which we can conclude that the interval of variation of these data has decreased. Six months post-intervention, the boundaries ranged from 11.00 to 32.29 mIU/mL, while at 12 months post-intervention, these values were 10.03 to 37.24 mIU/mL (Fig .2).

**Fig.3:**
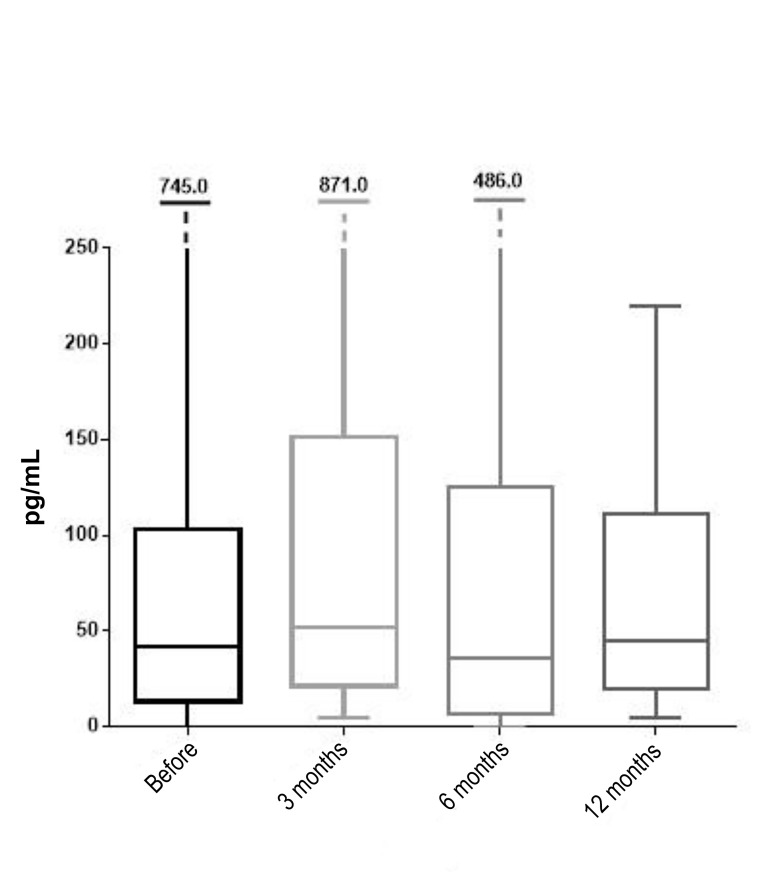
Medians, interquartile range and range of estradiol (E2) before the procedure and 3, 6 and 12 months after the procedure (line inside the box is median; the box is interquartile range; the lines outside the box are minimum and maximum values).

The third hormone observed in this study, E2, had a preintervention median value of 41.55 pg/mL, while 50% of the women had the value of this hormone in the range of 13.21 to 103.00 pg/mL. These values were obtained from a sample of 42 women who were evaluated for this hormone before intervention. From the observed 42 women, 24 were measured for E2 at 3 months post-intervention, when the median value of 52.38 pg/mL was recorded (26.6% increase compared to pre-intervention). The range of E2 hormone value was from 21.34 to 151.60 pg/mL. At 6 months following the intervention, the median value of the observed hormone was decreased to 36.00 pg/ mL (13.35% reduction compared to the pre-intervention value), from a sample of 22 women. The increase in the median value of E2 was recorded at 12 months post-intervention, when it was 45.10 pg/mL (8.54% increase compared to the pre-intervention value). This value was obtained from a sample of 19 women due to the loss of the follow-up measurements for this hormone (Fig .3). 

**Fig.4:**
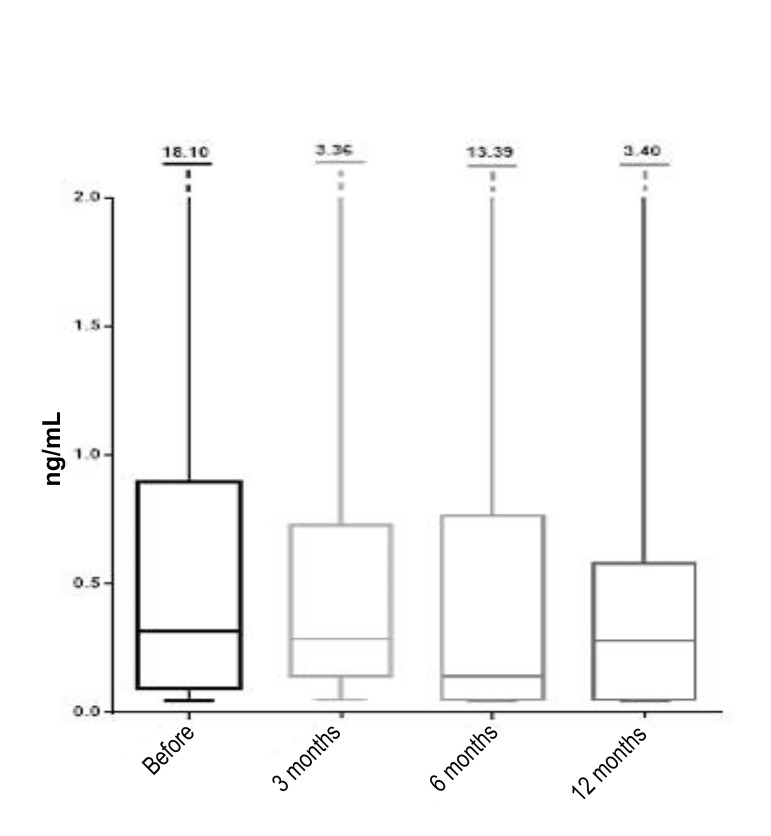
Medians, interquartile range and range of progesterone (PG) before the procedure and after 3, 6, and 12 months (line inside the box is median; the box is interquartile range; the lines outside the box are minimum and maximum values).

The hormone analysis also included follow-up measurements of PG levels in the subjects. Prior to the intervention, this hormone was measured in 32 women and its median value was 0.32 ng/mL. Half of the observed women had the value of this hormone in the interval from 0.09 to 0.89 ng/mL. After 3 months, the median PG value was 0.28 ng/mL (12.5% reduction compared to the value before intervention), while the intervals ranged from 0.14 to 0.73 ng/mL. These values were obtained from a sample of 12 women. At 6 months post-intervention, a decrease was observed in the median value of progesterone, being at 0.14 ng/mL (56.25% reduction compared to pre-intervention) based on the result of 13 women. The borderline intervals were, however, almost unchanged and ranged from 0.05 to 0.76 ng/mL. At 12 months post-intervention, based on the values measured from 11 women, the calculated PG median value was 0.28 ng/ mL (12.5% reduction compared with the value obtained before intervention), with 50% of the women having the value of PG in the range from 0.05 to 0.58 ng/mL (Fig .4). 

#### Changes in follicle stimulating hormone and luteinizing hormone hormonal levels before and after the intervention

The first aim of the study was to examine the differences in the levels of four different hormones and comparing their levels before and at 3, 6 and 12 months after ovarian intervention. Consequently, for each of the individual hormones, a null hypothesis has been tested that the hormone levels prior to and after the intervention are not different. This hypothesis was tested against the alternative hypothesis that differences in hormone levels do exist. Our results indicated that differences observed in FSH hormone levels in 19 women prior to the intervention compared to 3 months post-intervention are not statistically significant, since the value of Z statistics is -0.283 with a corresponding P value of 0.777. However, when the FSH hormone values were analyzed in 21 women 6 months after the intervention, it was noted that there were statistically significant differences in hormone levels compared to the pre-intervention values, with Z statistics having a value of -2.091, and the corresponding P value as (0.037). At 12 months post-intervention the level of the hormone observed in a sample of 13 women showed an insignificant difference compared to the level of the hormone before the intervention, with the P value of 0.196 with Z statistics -1.293 (data not shown).

With regards to the hormone LH, the results show that in 18 women there is not a significant difference between hormone levels at 3 months post-intervention compared to the pre-intervention LH levels, since Z is equal to -1.285 with a correspondinge P value of 0.199. Subsequently, the level of this hormone was measured and compared at 6 and 12 months post-intervention. After 6 months, 20 women were evaluated for the level of hormone LH in the blood and the results showed that there were no statistically significant differences in relation to its pre-intervention levels (Z=-0.443 with a corresponding P value of 0.658). The results for 15 women who were assessed for the level of this hormone at 12 months post-intervention were Z=-1.590 with a P value of 0.112, which was not significantly different compared to the pre-intervention measurements, at the significance level α = 0.10 (data not shown). 

The blood levels of E2 at 3 and 6 months post-intervention were measured in a sample of 21 women, displaying no statistically significant differences in the levels of this hormone before and after the intervention. The Z value of the statistics for comparing the differences between E2 levels before and at 3 months after intervention -1,195 with the corresponding P value of 0,232, which clearly indicates that significant differences do not exist, confirming the null hypothesis. After 6 months, the result of statistical analysis is the same [Z=-0.408 (P=0.683)], showing no significant differences between the levels before and after the intervention. After 12 months, 15 women were reevaluated for the E2 hormone and statistical analysis indicated that there were no significant differences in hormone levels before the intervention and 12 months after the intervention. The hormone PG was measured in a much smaller sample of women compared to the previously analyzed hormones; 8 women were evaluated at 3 months after the intervention, 10 women at 6 months after the intervention, and 7 women at 12 months post-intervention. It could be noticed that the differences in hormone levels prior to the intervention and 3 months after the intervention were not statistically significant, because the Z statistics was -0.845, and the corresponding P value was 0.398, therefore these findings suggested that the null hypothesis was correct. A null hypothesis was also adopted that there was no difference between the levels of hormone PG before and 6 months post-intervention, since the P value (0.735) that was corresponding to the Z statistics (0.338) was greater than α=0.10. When it comes to the differences in hormone levels of PG prior to and 12 months after the intervention, it is also concluded that these differences are not statistically significant due to a corresponding P value of 0.500, which is again greater than the level of significance of 10%.

#### Correlation between the re-transplanted tissue and hormonal excretion levels 

There are several factors affecting graft function after ovarian re-transplantation. The ovarian reserve in most of the patients re-transplanted with ovarian tissue is usually low due to the limited primordial follicles, so most of the pregnancies are conceived within the first 12 months after auto-transplantation. However, the main disadvantage is the limited number of available tissue fragments to be transplanted, due to ovary size. 

One of the aims of this study was to examine whether the volume of the autografted ovarian cortex affects the endocrine function of the ovary. The differences in the concentrations of the hormones FSH, LH, E2 and PG before and at 3, 6 and 12 months after transplantation were tested for correlation with the volume of the transplanted ovarian tissue (data not shown). Another parameter tested for a correlation between the differences in the concentrations of the hormones FSH, LH, E2 and PG before and at 3, 6 and 12 months post-transplantation, was the TNC from BMAC (Table 1). Our aim was to determine if a certain amount of stem cells are affecting the changes in the endocrine function of the ovarian tissue after re-transplantation. Multiple Spearman’s correlation analysis showed that significant correlation was found between the changes in FSH at 3 months post-transplantation and the volume of BMAC (P<0.088). The volume of BMAC was also correlated significantly with changes in PG after 3 months (P<0.092). TNC also correlated significantly with the changes in E2 levels at 12 months post-transplantation (P<0.088).

#### Ultrasound examination and monitoring of the follicular development

Close monitoring of ovarian function was conducted after re-transplantation, including repeated measurements of FSH and E2, PG and LH levels at 3, 6 and 12 months post-intervention, and continuous sonographic evaluations during 12 months. AMH levels were not good predictors of graft function, so they were not measured after transplantation.

Repeated ultrasound examinations were performed in patients by a single physician to clearly monitor follicle development from each ovary separately. Table 2 presents a summary of the total results of ultrasonography for all 50 patients. During the 12 months period after the re-transplantation 64% of patients had presence of a follicles. 

**Table 1:** Correlation analysis of the change in hormone levels (FSH, LH, E2 and PG) prior to and 3, 6 and 12 months post-transplantation, with the TNC from BMAC, and the volume of BMAC


Hormone	Time	Hormone change vs. BMAC volume P value	Hormone change vs. TNC P value

FSH	Change after 3 months	0.088	0.488
	Change after 6 months	0.843	0.641
	Change after 12 months	0.830	0.862
LH	Change after 3 months	0.314	0.229
	Change after 6 months	0.551	0.301
	Change after 12 months	0.665	0.713
E2	Change after 3 months	0.466	0.746
	Change after 6 months	0.183	0.599
	Change after 12 months	0.510	0.088
PG	Change after 3 months	0.092	0.391
	Change after 6 months	1.00	0.493
	Change after 12 months	1.00	0.670


These were tested by Spearman’s correlation, and considered significant at α=0.1. Values in the table represent P values. FSH; Follicle stimulating hormone, LH; Luteinizing hormone, E2; Estradiol, PG; Progesterone, TNC; Total nucleated cell count, and BMAC; Bone marrow aspirate concentrate.

**Table 2:** Monitoring of follicular development. Percentages in the table were given out of total number of patients


Total number of patients with follow-up 50	Egg cells total number	Embryos total number	Embryo transfer total number	Vitrified embryos total number	Number of newborns

32 Women (64% out of total number of patients) had Follicles	24	15	9	10	4
	8 Women (16% out of total number of patients) had eggs	6 Women (12% out of total number of patients) had embryos	4 Women (8% out of total number of patients) had ET	3 Women (6% out of total number of patients) had freezed embryos	3 Woman were pregnant (6% out of total number of patients)
Successful rates	25% Follicle positive women had eggs	75% egg positive women had embryos	66.6% embryo positive women had embryo transfer	50% embryo positive women had vitrified embryos	75% women with embryo transfers resulted with successful pregnancies
Follicles total noumber					231


Attempts to perform oocyte retrieval resulted in aspirated oocytes in 25% of the follicle-positive women (16% of the total number of patients). Fertilization rate of the aspirated oocytes was 75%, resulting in embryos in 12% of the women out of the total number of patients. Embryo transfers were performed in 66.6% of embryo-positive women (8% of the total number of patients), while 50% of embryo-positive women had vitrified embryos (6% of the total number of patients). Two patients spontaneously conceived after transplantation, while one pregnancy was conceived with IVF, resulting in the birth of twin babies.

### Discussion

In this study, we examined the effects of autologous *in vitro* ovarian activation using stem cells and autologous growth factors on reproductive and endocrine functions in patients with ovarian insufficiency. The results of the current study indicate that the SEGOVA procedure can play a potentially important role in addressing the problem of infertility in patients with impaired or lost ovarian function, as well as improving endocrine ovarian function, which affects a woman’s overall health and quality of life. With regards to the recent published data in the literature, the results of our study make important contribution to today’s scientific findings in the field of female reproduction, and open novel possibilities in treatment of female infertility. 

The profound socioeconomic changes in our society have increasingly caused women to delay the decision to start a family. Today, this factor leads to a major problem in the reproductive field, since female age is a decisive cause of infertility, it is common knowledge that as a woman gets older, both the quality and the number of her eggs available decrease. Therefore, many centers around the world are trying to address this problem by using different strategies to preserve fertility at an earlier age by for instance vitrification of the oocyte or using different therapeutic alternatives, allowing the patients to enjoy motherhood that would have been impossible in the past with low follicular reserve. 

The widespread use of oocyte donation is also a solution, but this is only a partial solution because many couples have serious difficulties in accepting such ideas. Therefore, preventive and therapeutic measures should be implemented. Preventive measures should address two relevant phenomena associated with ovarian aging: decreased follicles and decreased quality of oocytes contained within these follicles. Accordingly, ovarian aging has become a key challenge for reproductive medicine, as the ovaries change chronologically before other organs, causing fertility decline in the thirties, leading to ovarian fibrosis and complete loss of ovarian function in the early fifties (9-30). Advanced age, which affects both the quantity and quality of oocytes, became a major determinant of fertility (9). In the case of ovarian insufficiency, such as poor ovarian response and reduced ovarian reserve or primary ovarian failure, there remains a need for methods to restore fertility in patients seeking reproductive success (10). 

Due to the importance of aging in infertility, over the last 10 years, new research has emerged aimed at developing methods for rejuvenating oocytes by repairing genetic damage or introducing new sources of energy (11). Another source of healthy oocytes could be creation of gametes through the cell programing that could be expected in the future (12-14). The new potential egg source will pose a major challenge to the central dogma of reproductive biology, with which females of most mammalian species, including humans, lose their ability to create oocytes during fetal development. To date, recovery of ovarian function has been reported in preclinical models as well as clinical models using adult stem cells (12). MSCs ability to implant, survive, and reproduce in the ovaries was first evaluated by Liu et al. using mouse models after chemotherapy with damaged ovaries, where short-term fertility recovery and live births of healthy offspring have been reported (16, 17). 

Cord blood, amniotic membrane, menstrual blood, adipose tissue and endometrial tissue are considered to be possible sources of MSCs with promising results for several degenerative diseases within and outside the reproductive system (16, 17, 21, 22). Bone marrow is also an important source for mesenchymal cells, but their clinical use still requires improvements in culturing techniques to obtain adequate numbers of therapeutic cells. Today *in vitro* cultures increase the risk of cells losing some of their specific regenerative properties or accumulating chromosomal aberrations, which is also one of the reasons that we transferred BMSCs to the ovaries on the third day of the procedure, shortly after they were isolated from the bone marrow. To circumvent this concern, it was proposed in our study (SEGOVA) to use minimally invasive protocols, where bone marrow stem cells are not incubated under in vitro conditions and are rather under the control of ultrasound. These cells are then applied to ovarian tissue together with *in vitro* activated and incubated ovarian tissue with autologous growth factors. Here we have been able to regenerate the ovary in women with impaired or lost ovarian function. Based on this, we sought to evaluate the effects of autologous in vitro activation of ovaries following the transfer of growth factors and stem cells (SEGOVA) to the ovarian reserve in women with very poor prognosis. This novel study showed that SEGOVA improves ovarian reserve biomarkers and reproduction results, leading to the development of more follicles and oocytes after ovarian stimulation. This technique allowed for spontaneous pregnancies in women with POF diagnosis. In short, ovarian rejuvenation is a difficult task because age is characterized by a number of significant changes, including genomic instability, telomeric shortening, mitochondrial dysfunction and epigenetic changes (28). Addressing one of these questions may not be enough, thus we are working hard in this area of research and we hope to be able to present new important data soon. The technique which researchers call "*in vitro activation*", or IVA, requires the ovary (or part of the ovary) to be laparoscopically removed and treated outside the body and then laparoscopically reimplanted near its fallopian tubes.

After *in vitro* ovarian activation and re-transplantation of the activated tissue, a woman undergoes ovulation stimulation and undergoes IVF procedures. Follicular growth was observed in eight women, all of whom had signs of retained follicles before transplantation. These eight patients underwent ovulation stimulation, with five women developing mature eggs for IVF. The oocytes were fertilized with sperm from a male partner, and the resulting embryos were frozen and then transferred to the uterus. During the study, one patient underwent embryo transfer of one embryo, but failed to become pregnant, for another patient successful embryo transfer and pregnancy were achieved, but that pregnancy ended with a miscarriage (missed ab). The third patient underwent embryo transfer of two embryos, and had a successful pregnancy that resulted in the birth of a healthy boy. The remaining two women were preparing for embryo transfer and undergoing additional egg collection cycles. Since some of the patients had unsuccessful IVF or intracytoplasmic sperm injection (ICSI) attempts in the past, and on the other hand, some of them who had ovarian insufficiency did not have any IVF attempts before the SEGOVA procedure, the results of our study are very promising. The main limitation of this prospective clinical study is that we are not able to have a control group of females since the laparoscopic treatment would be required for isolation of ovarian tissue and its placebo treatment. However, at the same time avoiding the second laparoscopy and performing simple re-implantation of the activated ovarian tissue by ovarian aspiration needle under control of transvaginal ultrasound is a less invasive method, as performed in the past. A future perspective would be in exploring ways to control Hippo and phosphatase and tensin homolog (PTEN) pathways with drugs, without *in vitro* ovarian activation.

### Conclusion

SEGOVA ovarian rejuvenation procedure is unique because it uses the minimally invasive procedure of LPSC NOS surgery for taking a segment of the ovarian cortex for in vitro activation. Success criterion for this study was regaining the hormonal function in female subjects, activation of dormant follicles, promotion of antral follicle growth and development to mature oocytes. Another advantage of this method is that the transplantation of the activated tissue is performed under ultrasound control and not through laparoscopy. In SEGOVA PRP process there are also special systems and machines for separating certain cell lines, allowing to increase the concentration of desired cells (growth factors derived from them) up to 18 times the initial concentration. This approach is different from most other PRP ovarian therapies. While autologous BMSC transplantation can have a positive effect on patients with POF, allogeneic BMSC transplantation in women with POF can cause transplant rejection with further complications and consequences. SEGOVA acts on the intracellular signaling system and BMSC transplantation is without previous culturing and incubation in a way to save the original stem cell niche. The main difficulty with stem cell therapy is to maintain cell viability, cell properties and cell function before and after implantation *in vivo*. When stem cells are isolated from native tissue and grown and incubated in substrates, they rapidly lose their role and function that they originally had. In addition, they may have a shorter lifespan due to overexpansion *in vitro*. Furthermore, cellular DNA becomes unstable during long-term culture. Such hostincorporated cells lead to low cell survival rates and poor outcomes in growth, localization, differentiation, and paracrine effects. SEGOVA program overcomes these problems by performing autologous stem cell therapy without incubation. Within the study population, we showed that the hormone levels were different 6 months after the intervention, and it was noted that there were statistically significant differences among participants with respect to the level of the same hormone before the intervention.
